# Integrative diagnostics: the time is now—a report from the International Society for Strategic Studies in Radiology

**DOI:** 10.1186/s13244-023-01379-9

**Published:** 2023-03-30

**Authors:** Norman J. Beauchamp, R. Nick Bryan, Marilyn M. Bui, Gabriel P. Krestin, Geraldine B. McGinty, Carolyn C. Meltzer, Michael Neumaier

**Affiliations:** 1 Michigan State University, East Lansing, MI USA; 2 University of Pennsylvania, 3400 Spruce Street, Philadelphia, PA 19104 USA; 3 Moffitt Cancer Center and Research Institute, Morsani College of Medicine, University of South Florida, Tampa, FL USA; 4 Erasmus MC, University Medical Center Rotterdam, Rotterdam, the Netherlands; 5 Weill Cornell Medicine, New York, NY USA; 6 Keck School of Medicine, University of Southern California, Los Angeles, CA USA; 7 Medical Faculty Mannheim, University of Heidelberg, Mannheim, Germany

**Keywords:** Diagnostics, Radiology, Pathology, Informatics

## Abstract

**Abstract:**

Enormous recent progress in diagnostic testing can enable more accurate diagnosis and improved clinical outcomes. Yet these tests are increasingly challenging and frustrating; the volume and diversity of results may overwhelm the diagnostic acumen of even the most dedicated and experienced clinician. Because they are gathered and processed within the “silo” of each diagnostic discipline, diagnostic data are fragmented, and the electronic health record does little to synthesize new and existing data into usable information. Therefore, despite great promise, diagnoses may still be incorrect, delayed, or never made. Integrative diagnostics represents a vision for the future, wherein diagnostic data, together with clinical data from the electronic health record, are aggregated and contextualized by informatics tools to direct clinical action. Integrative diagnostics has the potential to identify correct therapies more quickly, modify treatment when appropriate, and terminate treatment when not effective, ultimately decreasing morbidity, improving outcomes, and avoiding unnecessary costs. Radiology, laboratory medicine, and pathology already play major roles in medical diagnostics. Our specialties can increase the value of our examinations by taking a holistic approach to their selection, interpretation, and application to the patient’s care pathway. We have the means and rationale to incorporate integrative diagnostics into our specialties and guide its implementation in clinical practice.

**Graphic abstract:**

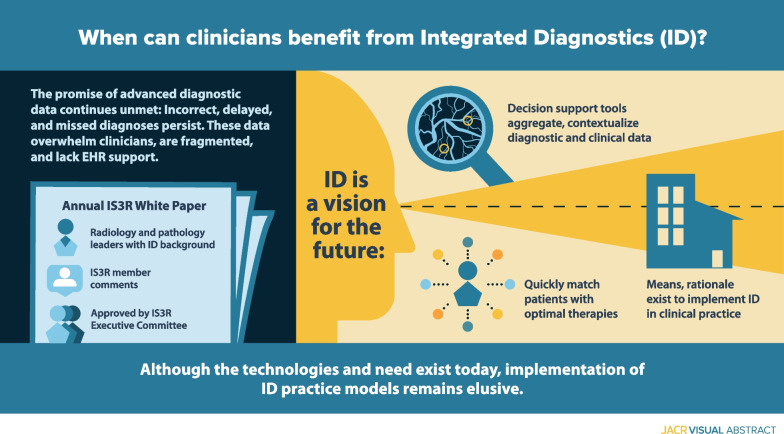

## Key points

- Although an overall boon to clinical diagnosis, increasingly voluminous, diverse, and fragmented diagnostic data can overwhelm physicians and frustrate patients.
- Data are gathered and processed within “silos” of the diagnostic disciplines, including radiology and pathology. The EHR does little to intelligently organize and synthesize these disparate data to facilitate diagnosis.
- ID envisions a process in which data from the entire arsenal of in vivo and in vitro diagnostics, together with clinical data from the EHR, are aggregated and contextualized to enhance diagnosis and direct clinical action

## Background

Despite digital imaging, widespread adoption of electronic health records (EHRs), and advances in precision medicine tools, diagnosis often remains a fragmented and frustrating process for clinicians and patients. Data are still gathered and presented asynchronously, and EHRs do little to organize and synthesize information. Although team practice, such as tumor boards, is increasing, routine physician interaction is limited by clinical workflow, high volumes, and IT boundaries. Despite an abundance of relevant diagnostic data, diagnoses may be incorrect, delayed, or never made. Allegations of diagnostic errors account for 28% of malpractice cases in the United States [1]. Experts estimate a diagnostic error rate of 10% to 15%, with 40,000–80,000 preventable deaths each year [2, 3]. As physicians and diagnosticians, it is our responsibility to minimize these errors. Integrative diagnostics (ID) has been proposed as one means to reduce diagnostic errors.

## Methods

This white paper is designed to address ID from the perspectives of radiology and our sister diagnostic specialty, pathology. The paper was developed in response to a request for proposal from the International Society of Strategic Studies in Radiology (IS3R) to its members for an annual white paper to foster its mission “to actively shape the future of medical imaging and image-guided therapies by leveraging the knowledge and influence of world leaders in these disciplines and related industries.” Proposals from self-organized writing groups were reviewed by the IS3R Publications Committee, with the final selection approved for drafting by the IS3R Executive Committee. Our writing group was designed to include departmental and institutional leaders in radiology and pathology who have interest and experience in ID. After preliminary approval by the Publications Committee, the draft paper was posted to the entire IS3R membership for comments, which were incorporated into this final document. This white paper was approved for internal dissemination and publication by the IS3R Executive Committee.

### What Is ID?

More than 7 billion diagnostic examinations are performed each year in the United States, influencing 70% of health care decisions [4]. Although diagnostic tests differ in personnel, infrastructure, and technology, they have a shared commonality: providing data for clinical diagnosis [5]. ID has been proposed to better manage, organize, and present diagnostic data and bridge intellectual silos. ID represents a convergence of imaging, pathology, and clinical laboratory medicine, plus advanced IT [6]. In this framework, integrated (versus isolated) practices plus clinical decision support (CDS) tools drive appropriate care. Data from the entire diagnostic arsenal are aggregated to enhance insights, and EHRs present information in a consumable way to facilitate collaborative decision making and accurate clinical diagnosis. ID uses medical informatics (in which data are data, regardless of their nature or source) to organize and analyze vast, disparate diagnostic data sets to achieve timely and accurate diagnosis, precise therapeutics, accurate assessment of prognosis, and maintenance of population health [7].

**Fig. 1:**
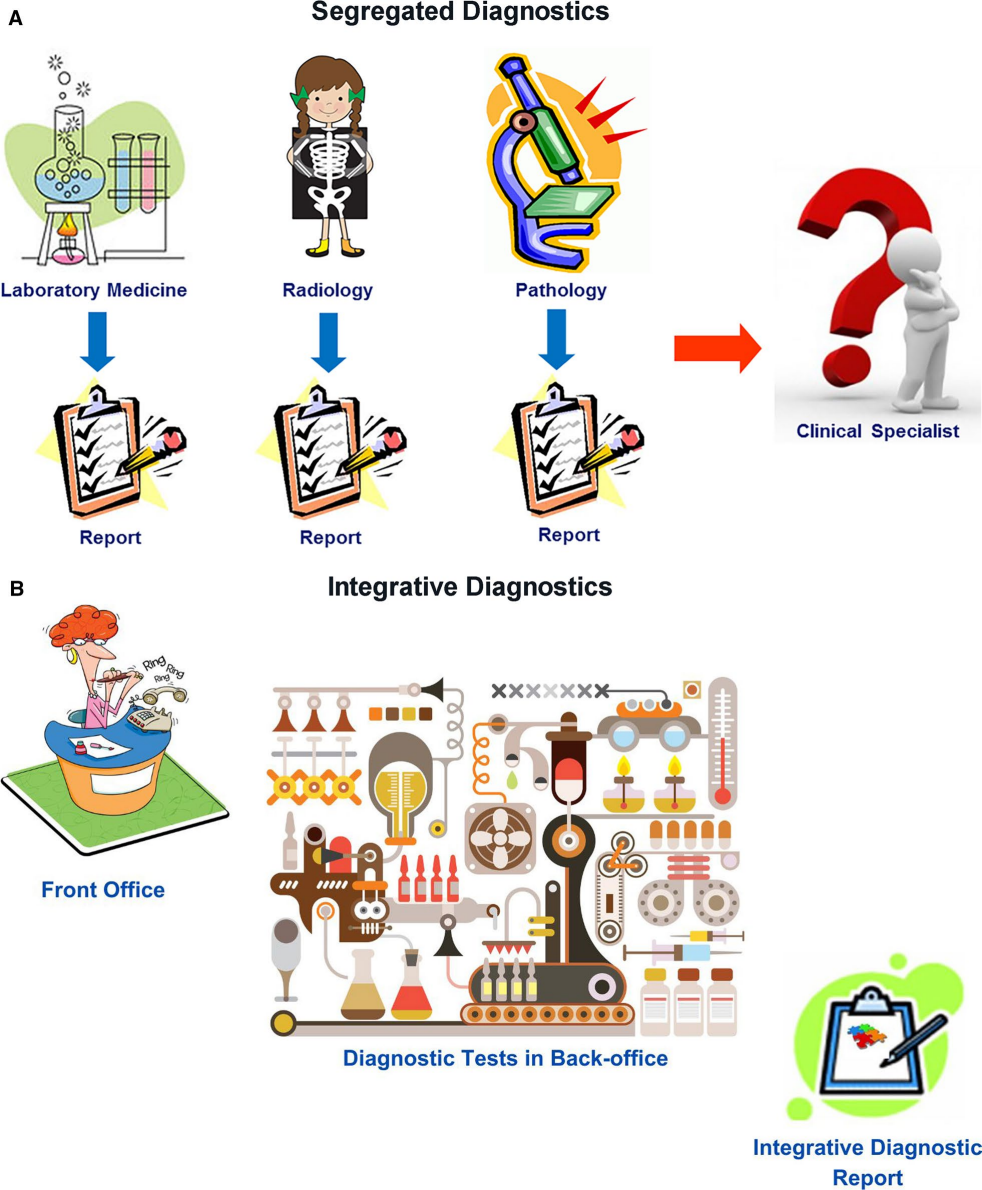
Diagram describing (**A**) segregated diagnostics versus (**B**) integrative diagnostics

Radiology, clinical laboratories, and pathology departments, which perform the preponderance of diagnostic tests, currently play a central role in medical diagnostics. However, our disciplines have not worked as an integrated unit. Rather, we are islands of vast data and extraordinary intradisciplinary expertise separated from one another and from our clinical colleagues by informatics, physical, and specialty barriers. We have not integrated our data or communicated them in a coordinated fashion to our clinical colleagues, instead expecting clinicians to integrate and interpret these data themselves. Although of immense potential value, our petabytes of data are increasingly overwhelming providers and systems as we “throw our work over the fence” and hope that someone figures out what it all means (Fig. 1A). It is no longer possible for individual health care providers to perform this complex task. ID offers a helping hand.

In ID, radiologists, pathologists, and other diagnosticians work as teams with shared access to continuously updated patient data, from which experts and CDS tools extract relevant clinical information and formulate dynamic differential diagnosis and management pathways (Fig. 1B). Given our in-depth knowledge of our test data, understanding of the pathobiochemical and physiological basis of our diagnostic findings, technological skills, and strong informatics resources and expertise, radiology and pathology should strive for leadership roles in the ID environment.

Predictive analytic tools based on aggregated clinical data can streamline care pathways so that appropriate diagnostic tests (including those performed by radiology, laboratory medicine, and pathology) are expedited on the basis of reason for referral, even in advance of a patient’s visit with a provider. This requires real-time data entry from all sources, continual analytics, and timely interactive communication among laboratories, providers, and patients. Triaging patients in this manner could streamline and more appropriately prioritize health care access. For example, by identifying patients who need to be seen sooner, a decrease in wait times for specialists would provide reassurance to patients earlier in their care journey and prevent them from turning to high-cost settings such as the emergency department for care. ID could direct patients to the correct therapy sooner, modify treatment when appropriate and terminate it when not effective, ultimately decreasing morbidity, improving outcomes, and avoiding unnecessary cost. Earlier access and more appropriate care are increasingly rewarded in value-based care payment arrangements. Additionally, ID could assess information that affects both individual patient well-being and population health, including identification of emerging infections, antibiotic resistance, exposure to toxic substances, and chemical or biologic threats.

Despite a clear need and sound theoretical reasons for expanding the role of radiologists and pathologists in ID, real-world efforts remain meager. Our purpose is to stimulate more ID activity in our specialties by presenting the rationale for such efforts, highlighting successful ID programs that might be emulated at other sites, and recommending specific endeavors that are feasible now and should be prioritized in our departments and institutions.

### The ID process

**Fig. 2:**
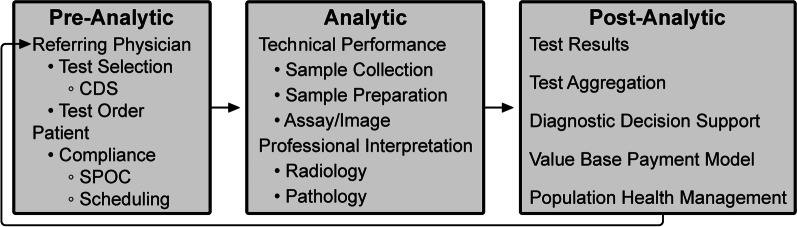
Integrative diagnostic workflow phases. *CDS*  clinical decision support; *SPOC*  single point of contact

As outlined by the Institute of Medicine Committee on Diagnostic Error in Health Care, the diagnostic examination process is divided into three phases: pre-analytic, analytic, and post-analytic [7, 8] (Fig. 2).

The analytic phase is the least susceptible to errors because of attention to technical performance and procedural standards, rigorous internal management and external quality assessment, and precise quantitative measurements. In contrast, our relative inattention to the pre- and post-analytic phases now warrants modification. In laboratory medicine, the analytic phase accounts for approximately 25% of total effort and workflow, the pre-analytic phase for 57%, and the post-analytic phase for 17% [9]. A disease process that requires inputs from multiple diagnostic disciplines is typically interrogated in a stepwise and discontinuous way. Although this is sometimes unavoidable (including subsequent testing whose utility only becomes apparent on the basis of preceding tests), the fragmented, sequential nature of the diagnostic process can cause treatment delays with negative impacts on outcomes [10]. ID can accelerate medical diagnosis, transforming this discontinuous, slow, and fragmented approach into a highly coordinated process with faster information flow through these test phases.

In the pre-examination phase, the referring provider is responsible for performing and/or requesting the most appropriate examinations. In an estimated 10 to 15% of cases, the referring provider needs, and would value, assistance with these decisions. Inappropriate laboratory testing involves both under- and overutilization and occurs in 20 to 30% of cases [11]. Extra support may be needed for primary care providers, who see a wide variety of patients and diseases [12]. Modern health care informatics can help providers request the most appropriate examinations by integrating CDS tools into clinical workflow [13]. For example, use of a real-time radiology appropriateness CDS application decreased inappropriate utilization of brain and spine MRI and sinus CT [14], and computer-generated reminders to clinicians in Kenya improved CD4 laboratory monitoring of patients with HIV infection [15]. CDS tools should be available on platforms that integrate seamlessly into providers’ workflow without adding unnecessary steps, and the appropriate use criteria underpinning these tools must be evidence based. Feedback to providers must be supportive and advance learning, rather than being punitive. Additionally, CDS systems should be adapted to local circumstances, including local patient demographics and resources (such as the availability of diagnostic test equipment and the competencies of diagnosticians). CDS systems must also be capable of incorporating all relevant patient data. Moreover, CDS should be “integrated,” reflecting not only the appropriateness of a single diagnostic discipline but also the benefit of combinations of tests across disciplines.

Although referring physicians are responsible for maximizing the likelihood that patients will get needed examinations, approximately 20% of requested examinations in the United States are never performed [16]. Well-designed health care systems using contemporary, web-connected logistic support tools can improve this by coordinating examination times across disciplines at sites that match patients’ circumstances and preferences to local health care resources. Point-of-care (POC) testing and service increases patient test completion, satisfaction, and clinical outcomes, although it presents efficiency and quality control challenges. POC laboratory testing has improved clinical outcomes in influenza and pneumonia, HIV infection, heart attack, and strep throat [17]. In Berlin, the use of mobile stroke units with CT scans and POC laboratory tests resulted in decreased time to treatment and lower global disability at 3-month follow-up [18].

The analytic phase of the diagnostic process centers on the performance of each specific examination, which are currently relatively independent events. Most PACS and radiology information systems are separate from laboratory information systems, resulting in each pathologist and radiologist interpreting their own studies without easy access to the others’ results. Bridging this disconnect should be a radiology and pathology IT priority. Diagnostic accuracy and management recommendations are improved when examinations are tailored and interpreted using knowledge from previous tests. Modern informatics, through optimizing the EHR, should make the complete medical record available to every examiner at the time of an examination, along with appropriate management guidelines. Each study’s unique information should be intelligently and intuitively encapsulated in each sequential result. For example, POC CDS for management of incidental lung nodules improved adherence to nationally recommended guidelines for follow-up [19].

The post-analytic phase of diagnostics initially focuses on the application of test results to the individual patient’s diagnosis and care plan. The aggregation and diagnostic inferences from all of a patient’s examinations leads to the most accurate and specific diagnosis. Examination results should be promptly and intuitively incorporated into the EHR and made easily available to health care providers and the patient. Until the past decade, these medical data were analyzed by a single or small number of providers, primarily using heuristics. Although an invaluable human thought process, heuristics takes shortcuts in reasoning, may not use all available data, and has well-known sources of error, including cognitive, selective, and availability biases [20]. Furthermore, heuristics suffers reduced accuracy and efficiency with increasing volumes and diversity of data types and greater task complexity.

Deficiencies in current practice and EHRs extend from the most basic error—missing data—to data overload, as with radiopathogenomics. For example, in a Veterans Affairs setting, 30% of providers reported encountering at least one patient with a missed test result over the previous 2 weeks that caused a delay in diagnosis or treatment [21]. Tumor boards, with their extensive clinical, imaging, laboratory, and anatomic pathology content, may represent the epitome of data overload. No single human can master all the information of even one patient in any reasonable period of time, which was the topic of an RSNA/American Association of Physicists in Medicine symposium on ID in 2019 [22]. The current explosion of remote health monitoring tools and diagnostic tests with exponentially larger data units can easily overwhelm a single astute physician. It is estimated that every patient generates 80 MB of data each year, and the volume of health care data is predicted to increase faster than any other business sector [23, 24]. ID can bring more human and computational resources to bear on these essentially raw data to yield useful information to diagnose and treat individual patient problems as well as address population disease and health management [25].

An intelligent informatics infrastructure leveraging all these individual and population data can greatly augment the traditional human analysis. Integrated structured reports with discrete data are critical for data aggregation, which, in conjunction with outcome data, allows the development and optimization of front-end CDS systems. CDS tools, incorporating artificial intelligence and machine learning methodology, can provide referring providers with real-time probabilistic differential diagnoses for individual patients and enable the development of management paradigms for specific diseases and large populations [26]. Unfortunately, many CDS systems are monodisciplinary, prematurely obsolete, and incompatible with efficient clinical workflow. Modern health care informatics must develop fully integrated CDS tools that are multidisciplinary, continuously updated, and adapted to the local situation. These new management paradigms can close the loop from the post-analytic back to the pre-analytic phase by suggesting the most appropriate examinations for the referring physician’s next patient with a similar problem set. Furthermore, this information can help health care systems identify their most burdensome and needy patients for proactive health care management.

In the United States, the 21st Century Cures Act emphasizes the patient’s role in the post-analytic phase. The program rule on interoperability, information blocking, and Office of National Health Coordinator health IT certification, which implements this act, requires that health care providers give patients access without charge to all the health information in their EHR “without delay” [27]. This legislative directive, predicated on evidence that optimal diagnosis and treatment are enhanced by the convenient availability of patients’ medical records to their physicians, will further drive the aggregation of medical information, regardless of initial source or current repository. This initiative should better enable all physicians involved in a patient’s care to have immediate access to all of that patient’s health care information. Although providing invaluable information to providers, including radiologists and pathologists, the technical and legal demands of this act on provider practices, health systems, and IT vendors will be significant. The requirement that all components of the EHR be promptly provided to the patient, including all laboratory, anatomic pathology, and radiology reports, presents a communication challenge and an opportunity for ID. We will have to modify our reports for patients’ consumption through health care portals, offering the opportunity to integrate and summarize test results for patients and referring physicians.

### ID and radiology and pathology: “In vivo” meets “In vitro”

In 2020, the European Society of Radiology and the European Federation of Laboratory Medicine signed a memorandum of understanding confirming international support of ID between both disciplines [28]. Underlying this alliance is the favorable complementarity of diagnostic scope and data generated by the two fields. Modern imaging technologies provide high-resolution morphologic information but limited information on tissue metabolism and potential function and no systems information. In contrast, clinical laboratory medicine measures thousands of biochemical and molecular markers with moderate to high tissue specificity in various bodily fluids, but it rarely gives the pinpoint morphologic information that radiology can provide. For example, molecular biomarkers can sensitively indicate the presence of minute brain lesions, but these markers cannot localize a defect within the organ, assess the size of a lesion, or even count the number of lesions, functions that are easily provided by high-resolution in vivo imaging [29].

Table 1 demonstrates the clinical potential of ID between imaging and laboratory medicine. Each diagnosis or medical condition requires the results of a specific set of clinical, laboratory, radiology, and pathology tests to achieve a precise diagnosis. To make efficient use of our siloed data for ID, we must define interdisciplinary biomarker sets for specific clinical indications. Currently, physicians order examinations or tests from different disciplines and integrate the data themselves. With ID, clinical questions can be addressed by “in vivo” and “in vitro” diagnostic medicine, with different disciplines integrating their respective data and reporting interpreted results to other providers and patients in a combined report. Now is the time for radiologists and pathologists to venture beyond our disciplines and engage the broader diagnostic challenges confronted by ID. In the “Recommendations” section, we provide concrete advice on integration of ID into current practices.

**Table 1:** Examples of integrative diagnostics

Disease	Clinical Findings	In Vivo Dx (Imaging)	In Vitro Dx (Laboratory Medicine)	In Vitro Dx (Pathology)	References
*Cancer*					
Prostate	DRE, osseous lesion	Transrectal ultrasound, MRI, PET	PSA, acid phosphatase		[40, 41]
Colorectal	Occult blood, ileus	CT, MRI, PET	CEA, CA 19–9, blood count	Histopathology, MSI assessment	[42]
Pancreatic	Jaundice	MRI	CA 19–9, bilirubin, GGT	Histopathology	[43]
Breast	Clinical examination	CT	CA 15–3	Histopathology, ER and PR status, HER2neu	[44]
Brain	Neurologic deficit	CT, MRI	S100β		[45]
Lung			CEA, SCC, NSE, CYFRA 21–1		[46]
Plasmocytoma	Spontaneous fractures	Whole-body MRI	Blood count, serum electrophoresis, IEF		[47, 48]
*Cardiovascular*					
Chest pain		Triple-rule-out ECG-gated CT	TnI/TnT, NT-proBNP, BNP, D-dimer		[49]
Dyspnea		Thorax	NT-proBNP, BNP, differential blood count, creatinine, GFR, CRP, PCT		[50]
*Neurological*					
Stroke	Hemiparesis	CT angiography, MRI	GFAP, S100β		[51]
Meningitis	Neurologic deficit, meningitis sign (eg, Kernig sign)		CSF cell count, albumin, IgG, IgA, IgM, glucose, lactate, bacteria, CRP, PCT, sepsis parameter, bacterial culture		[52]
Multiple sclerosis	Specific neurologic deficit		CSF cell count, albumin, IgG, IgA, IgM, glucose, CRP, oligoclonal bands		[53]
Encephalitis					
*Inflammation*					
Sepsis		Focus search	CRP, PCT, cytokine profiles IL-6, IL-8, differential blood count, blood culture		[54]
Rheumatoid arthritis		Bone imaging	CRP, sedimentation rate		[55, 56]
*Endocrinology*					
Addison or Conn syndrome	Hypertension/hypotension, cardiovascular symptoms, stress	MRI, selective adrenal venous sampling	Adrenal hormones, orthostasis test, salt exposure test		[57, 58]
Hypophysitis	Headache, altered vision, diabetes insipidus, signs of adrenal insufficiency	CT, MRI	Adrenal hormones, TSH, CRH, CRP, drugs and therapeutic medications		[59]
Multiple endocrine neoplasia syndromes				Mutation status: MEN, Ret, VHL1	
Diabetes	Polyuria, polydipsia, fatigue, weight change, CKD, retinopathy		Glucose, HbA_1c_, C-peptide, urine-Stix, blood gases and blood pH, lactate, ADH, copeptin, GADD45 antibodies, islet cell antibodies, IA-2 antibodies, insulin		[60, 61]
Hypothyroidism	Fatigue, weight gain, low activity, depression, blood pressure, HR	Thyroid ultrasound	TSH, FT4, FT3, Tg, TRAK and TPO antibodies		[62]
Congenital adrenal hyperplasia	Virilization, salt loss	Ultrasound	Cortisol, aldosterone, electrolytes	Karyotyping, mutation status: 21-OHase, 17a-OHase	[63]

*ADH* antidiuretic hormone, *BNP* B-type natriuretic peptide, *CA 15–3* cancer antigen, *CA 19–9* carbohydrate antigen, *CEA* carcinoembryonic antigen, *CKD* chronic kidney disease, *CRH* corticotropin-releasing hormone, *CRP* C-reactive protein, *CSF* cerebrospinal fluid, *CYFRA 21–1* cytokeratin fragment, *DRE* digital rectal examination, *Dx* diagnosis, *ECG* electrocardiography, *ER* estrogen receptor, *FT3* free T3, *FT4* free T4, *GADD45* growth arrest and DNA damage, *GFAP* glial fibrillary acidic protein, *GFR* glomerular filtration rate, *GGT* gamma-glutamyl transferase, *HbA*_*1c*_ glycated hemoglobin, *HER2neu* human epidermal growth factor receptor 2, *HR* heart rate, *IA-2* islet antigen, *IEF* isoelectric focusing, *Ig* immunoglobulin, *IL* interleukin, *MEN* multiple endocrine neoplasm, *MSI* multisatellite instability, *NSE* neuron-specific enolase, *NT-proBNP* N-terminal pro–B-type natriuretic peptide, *OHase* hydroxyvitamin hydroxylase, *PCT* procalcitonin, *PR* progesterone receptor, *PSA* prostate-specific antigen, *Ret* rearranged during transfection, *SCC* squamous cell carcinoma, *Tg* thyroglobulin, *TnI* troponin I, *TnT* troponin T, *TPO* thyroid peroxidase; *TRAK* thyrotropin receptor antibodies, *TSH* thyroid-stimulating hormone, *VHL* Von Hippel Lindau

### Why ID?

#### Added clinical value

Good medical practice demands that only clinically appropriate examinations be performed, with minimum achievable risk. Socioeconomics requires that medical examinations be performed in a cost-effective fashion, while minimizing discomfort or inconvenience. The search for value in health care spending often casts imaging and diagnostics as drivers of cost and wasteful overutilization. However, diagnostic examination results are directly or indirectly involved in approximately 70% of medical decision making, while requiring less than 3% of the money spent on health care expenditures. In an effort to ensure value, hospitals, payers, and regulatory agencies track cost and quality performance; ID has the potential to improve both. Predictive analytic tools based on aggregated clinical data can streamline care pathways so that appropriate imaging and diagnostics are prioritized and expedited, on the basis of continual asynchronous informatics tools operating outside of, but in parallel with, direct communications and visits with patient providers.

Our health care delivery system is rife with opportunity for streamlining care and reducing cost. We must find more cost-effective approaches to evaluating and bringing better health care not only to an individual patient but to our populations of patients, especially those who may have been historically disadvantaged. Since 2002, the AMA has emphasized the roles and responsibilities of physicians to promote the public’s health [30]. Patients in rural areas often experience barriers to health care, including radiology and laboratory services, that limit their ability to receive appropriate care. Not surprisingly, these deleterious effects are magnified for minority, underserved, and underdeveloped populations [31]. ID offers unique and necessary tools to address these broader social demands on health care.

Local reimbursement systems present formidable barriers to change, including the implementation of ID. The prevailing fee-for-service reimbursement system in the United States offers little incentive to pool intellectual and informatics resources to develop an ID approach. Regulatory constraints around fee sharing have been reduced by the accountable care and bundled payment programs (Obamacare), but these programs still represent a minority of health care reimbursement. Even in single-payer systems such as the United Kingdom’s National Health Service, cultural barriers may exist arising from the desire to maintain individual department resources. Importantly, the large capital investments in informatics infrastructure that will be required to manage a robust ID workflow could actually delay innovation when new, more integrated IT systems are needed in the future. The Health Information Technology for Economic and Clinical Health Act of 2009 to incentivize the adoption of EHRs included $27 billion to help finance the endeavor [32]. The current Cures Act does not have comparable governmental financial support. The lack of clarity on how the significant investment required of medical informatics companies will be rewarded represents another reimbursement-related barrier to innovation.

#### Discovery

Imaging and advanced molecular diagnostics have hastened the pace of discovery by providing quantitative outcome measures and decreasing subject variability in clinical trials, while lessening required sample size [33]. ID can expand our ability to perform efficient clinical trials with diverse patient populations. Using “real-world” data allows pragmatic research based on “computable phenotypes,” resulting in clinical cohorts from multiple sources containing data gathered in clinical care, home, or community settings [33]. It also enables more practical “pseudorandomized” clinical trials, which is important in a setting of decreased margins and demand for faster discovery. Furthermore, many diseases respond to multimodality therapy, and as such, evaluating single therapy interventions using traditional outcome measures and randomized clinical trials may miss therapies with important subclinical effects.

#### Medical education

ID principles should be formally introduced in the first year of medical school, beginning with the concepts of team medicine and differential diagnosis on the basis of Bayesian inference, and pathologists and radiologists should be active participants. The current organization of undergraduate and graduate medical education fosters departmental silos, compounding fragmented informatics and information infrastructure. Traditional teaching must be changed to reflect the ID workflow, as in the problem-oriented curricula adopted in the Netherlands [34]. Table 2 includes recommendations for ways to encourage ID in medical education.

**Table 2:** Recommendations

Organizational	Educational	Operational	Research
Expand multidisciplinary teams: “tumor boards” for all disciplines National organizational support Incorporate ID sessions into annual professional meetings Joint leadership meetings Cross-departmental professional and administrative structure Associate dean for ID Joint committee of radiology/pathology leadership	UME: Principles of diagnosis radiology, laboratory, and pathology instructors GME: Joint radiology and pathology presentations and conferences CME: Joint professional society courses	Integrate radiology and pathology IS (with EHR) Monitor and improve joint workflow Coordinated diagnostic test scheduling Increase POC testing ID turnaround time: test 1 to 2 to 3 to dx to rx Document and QC diagnostic errors Routine 360° follow-up for all diagnostic tests: missed examinations, missing reports; missing referral physician follow-up	Integrated reporting mechanisms Advanced CDS functions ID financial models Real-world trials for diagnostic tests

CDS clinical decision support, *CME* continuing medical education, *dx* diagnosis, *EHR* electronic health record, *GME* graduate medical education, *ID* integrative diagnostics, *IS* information systems, *POC* point-of-care, *QC* quality control, *rx* treatment, *UME* undergraduate medical education

### ID now?

Although separate radiology and pathology departments remain the norm at academic medical centers, early efforts at ID departments have been made. At Erasmus MC, University Medical Center Rotterdam, one of the largest academic hospitals in the Netherlands, clinical departments are organized in themes (G.P. Krestin, personal observation, 2022). The theme Diagnostics and Advice gathers all diagnostic departments (radiology and nuclear medicine, pathology, laboratory medicine, microbiology, virology, immunology, and pharmacy). The leadership of the theme consists of the department chairs and is committed to implementing the concept of ID to deliver high-quality ID reports to the referring physicians of the hospital. However, in the initial phase there was resistance from both clinical and diagnostic staff: referring clinicians considered that decision for the choice of diagnostic tests needed to remain their domain, while the diagnostic staff was reluctant to take on board additional “burdensome” exchanges with their colleagues in other diagnostic specialties. To break the deadlock, leadership started with a number of pilot cases (lung cancer, adrenal incidentaloma, primary liver lesions) that all turned out to benefit from an ID approach.

In 2018, the new Dell Medical School of the University of Texas at Austin established the Department of Diagnostic Medicine, which incorporated radiology and pathology (R.N. Bryan, personal observation, 2022). The organization of this department features a tripartite leadership of the chair and co-chairs or chiefs of clinical radiology and pathology. The chair has primary responsibility for the research program, while the clinical chiefs have primary responsibility for their respective clinical services. Responsibility for the educational programs is shared by these three departmental leaders, who are supported by appropriate vice chairs for education and research from radiology and pathology faculty members. Although still nascent, progress of the program is internally viewed as “encouraging.” Newly ACGME-credentialed radiology and pathology residency programs will accentuate radiology and pathology teaching conferences and multidisciplinary clinical conferences.

A major limitation to these joint efforts is IT infrastructure. At Erasmus MC, the recent digitization of the pathology department and use of similar image management systems in pathology and radiology is expected to further facilitate integration between these specialties and the health system EHR. At the Dell Medical School, IT remains separate and fragmented, not only between radiology and pathology but also with the hospital EHR and other specialty information systems. Although technological integration of IT infrastructure (radiology information systems, laboratory information systems, PACS, EHR, etc.) is necessary for the success of ID, it is not sufficient. Coordinated, multispecialty oversight of the ensemble is critical and must still address the specific needs of each specialty, while at the same time presenting a seamless overview to the clinical, research, and educational communities.

## Recommendations

Progress toward a more integrated approach to medical diagnostics has been slow, even in organizations that have created structures to foster it. Silos of expertise and incentives are deeply ingrained. Some might argue that it will take regulation or significant payment innovation to break them down. The digitization of pathology, pathology’s “third revolution,” and the application of artificial intelligence to medical data create a sense of both possibility and urgency around this effort. Key drivers of success will be organizational matrices that foster communication and collaboration supported by robust informatics infrastructure. A general model of this concept is the integrated practice unit (IPU), which defines a multidisciplinary team of appropriate clinical and patient support personnel to address the full care cycle of a patient condition, supported by necessary physical, financial, and IT elements [35]. A relatively unique view of the currently ill-defined roles of radiologists and pathologists in an IPU is that of an information specialist [36]. Here, the radiologist’s or pathologist’s responsibility is not just the extraction of information from images or histology but management of that information (plus information extracted by artificial intelligence) in the clinical context of the patient.

Change must begin with small, easy steps, such as preclinical ID presentations by radiologists and pathologists, more joint radiology and pathology teaching sessions in our graduate medical education programs, focused postgraduate training programs jointly sponsored by our professional organizations, and extension of the tumor board concept into other disciplines, such as heart failure and infectious disease. However, the initial smaller steps should culminate in an organization with the will to implement a strong ID program and a way to support it through robust informatics. Table 2 lists recommendations for changes, some more immediately “doable,” others more demanding but achievable. The previous section described early ID efforts at Erasmus MC and the Dell Medical School. A third example, the Center for Integrated Diagnostics at the Massachusetts General Hospital, with the mission “to foster development of clinical actionable diagnostics and accelerate the adoption of personalized medicine,” also recognizes the need to extend the concept beyond oncology and expand its testing to other disciplines [37].

The failure of widespread adoption of the IPU concept of disease-focused care does not bode well for the health care system’s ability to adapt [38]. Creating financial models that demonstrate the economic value proposition of ID will be a necessary catalyst for change. We believe creating a clear vision of the value created by ID for patient outcomes and quality of care will be most effective. Berwick’s “dimensions of total quality” are all served by ID: don’t kill me (no needless deaths from improper diagnosis); do help me (with a quick diagnosis); don’t hurt me (no needless pain or unnecessarily invasive tests); don’t make me feel helpless (inform patients of the reason and results of diagnostic tests); don’t keep me waiting (or running between multiple test sites); and don’t waste resources, mine or anyone else’s (perform the fewest, best, least expensive tests) [39].

## Data Availability

All relevant data is included in this work.

## Abbreviations

CDS: Clinical decision support
EHR: Electronic health record
ID: Integrative diagnostics
IS: Information systems
IS3R: International Society of Strategic Studies in Radiology
POC: Point-of-care
SPOC: Single point of contact
